# Foot Osteochondroses

**DOI:** 10.3390/children12091114

**Published:** 2025-08-24

**Authors:** Antonio Mazzotti, Gianmarco Gemini, Laura Langone, Alberto Arceri, Simone Ottavio Zielli, Federico Sgubbi, Gianmarco Di Paola, Maurizio De Pellegrin, Cesare Faldini

**Affiliations:** 1 Department of Biomedical and Neuromotor Sciences (DIBINEM), University of Bologna, 40127 Bologna, Italy; antonio.mazzotti@ior.it (A.M.); cesare.faldini@ior.it (C.F.); 2 1st Orthopaedics and Traumatologic Clinic, IRCCS Istituto Ortopedico Rizzoli, 40136 Bologna, Italy; gianmarco.gemini@ior.it (G.G.); laura.langone@ior.it (L.L.); simoneottavio.zielli@ior.it (S.O.Z.); federico.sgubbi@ior.it (F.S.); gianmarco.dipaola@ior.it (G.D.P.); 3 Orthopaedics and Traumatology, Papa Giovanni XXIII, 24127 Bergamo, Italy; depellegrin1956@gmail.com; 4 Pediatric Orthopedic Unit, Piccole Figlie Hospital, 43125 Parma, Italy

**Keywords:** tarsal bones, osteochondrosis, foot, pediatrics, osteochondritis, avascular necrosis, vascular supply, deformity, joints, cartilage

## Abstract

**Highlights:**

**What are the main findings?**

- Foot osteochondroses are rare, self-limiting, growth-related disorders that affect various ossification centers, most commonly the calcaneus, navicular, and metatarsals.
- This review presents a comprehensive anatomical mapping and description of foot osteochondroses.

**What is the implication of the main findings?**

- Despite being uncommon, early recognition of foot osteochondroses is essential to prevent long-term sequelae, such as deformities or secondary osteoarthritis.
- A shared conservative treatment approach—centered on rest, offloading, and symptom control—can often lead to favorable outcomes and avoid unnecessary surgical interventions.

**Abstract:**

Osteochondroses of the foot represent a unique and less frequently discussed topic. This narrative review aims to provide a comprehensive overview of foot osteochondroses, highlighting their definition, pathophysiology, clinical features, diagnosis, and treatment. Historical sources, including early case reports, were included along with the current literature to picture the current knowledge on the subject. Anatomical mapping of pain locations and associated ossification centers was employed as a framework to present the various forms of foot osteochondroses. Multiple types of foot osteochondrosis were identified. The calcaneus, navicular and lesser metatarsal are among the more common involved bones. Most forms share a multifactorial etiology involving mechanical stress, vascular insufficiency, and delayed ossification. The pain is localized and common to all forms. Diagnosis relies on clinical assessment supported by radiographic and sometimes magnetic resonance imaging findings. During the acute phase, joint rest is essential. Despite the potential for spontaneous resolution, some cases can lead to structural deformities or persistent symptoms. Foot osteochondroses, although rare, require careful clinical evaluation due to their impact on pediatric patients. Increased awareness and standardized treatment approaches may improve early recognition and management, potentially reducing long-term sequelae.

## 1. Introduction

Osteochondroses are heterogeneous growth-related and self-limiting disorders characterized by degenerative and necrotic processes affecting the endochondral ossification centers. The term osteochondrosis therefore differs from osteochondritis, as it refers to a non-inflammatory condition. Osteochondroses may involve epiphyseal, apophyseal, and some short bone ossification centers. The degenerative–necrotic processes are followed by reparative bone responses, which may or may not result in anatomical deformities [1].

Osteochondroses mainly affect male patients between the ages of 3 and 17 years. All ossification centers can potentially be involved, with the lower limbs being the most frequent site. In approximately 15% of cases, involvement is bilateral [2,3,4,5].

The pathogenesis of osteochondroses remains incompletely understood. While mechanical overload, vascular insufficiency, and repetitive microtrauma are commonly implicated, the precise etiology is likely multifactorial [6]. Other less widely accepted theories have also proposed etiologies of endocrine, metabolic, infectious, neurogenic, and congenital origin [7]. In the foot, osteochondroses represent a relatively rare but clinically relevant subset of the pathology, most often presenting with pain, limping, and functional impairment that prompt medical evaluation [8]. These disorders are associated with specific radiographic patterns and may show spontaneous resolution over time. Despite that, in some cases, they can lead to structural deformities or long-term sequelae [8]. For this reason, foot osteochondroses must be promptly diagnosed and treated in order to prevent complications in adolescence and adulthood, which may sometimes require invasive and destructive surgical intervention [7]. This latter possibility is particularly frequent in anatomical sites subjected to high weight-bearing loads, such as the foot.

This narrative review aims to provide a comprehensive overview of osteochondroses affecting the foot, with a focus on the most frequently involved anatomical sites, their definition, historical notes, epidemiology, pathophysiology, clinical features, diagnosis, and treatment.

## 2. Types of Foot Osteochondroses

**Figure 1:**
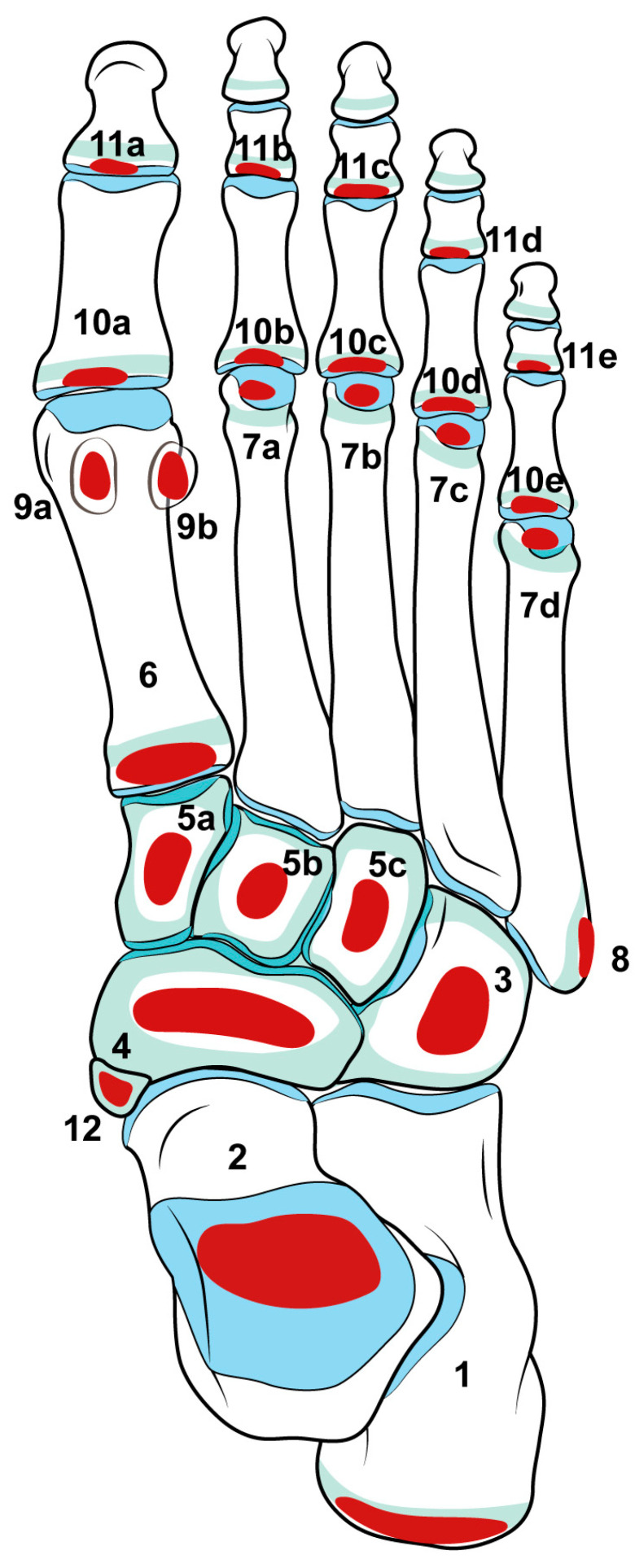
Foot osteochondrosis. 1: Calcaneal osteochondrosis. 2: Talar osteochondrosis. 3: Cuboid ostechondrosis. 4: Navicular osteochondrosis. 5: Medial (a), intermediate (b), and lateral (c), cuneiform bone osteochondrosis. 6: First metatarsal base osteochondrosis. 7: Lesser metatarsal head osteochondrosis (a–d). 8: Fifth metatarsal base osteochondrosis. 9: Medial (a) and lateral (b) sesamoid bone osteochondrosis. 10: Proximal phalange osteochondrosis (a–e). 11: Distal phalange osteochondrosis (a–e). 12: Accessory navicular osteochondrosis.

Figure 1 shows a map indicating all the possible foot osteochondrosis locations (Figure 1).

The following section will provide a comprehensive overview of the various osteochondroses affecting the foot, with particular emphasis on the epidemiology, pathophysiology, clinical presentation, and diagnostic approach for each condition. Therapeutic management will be addressed in a subsequent section, given the similarities in treatment strategies across the different anatomical sites.

### 2.1. Calcaneal Osteochondrosis

#### 2.1.1. Definition and Historical Notes

Calcaneal osteochondrosis is a common overuse condition affecting the growth plate (apophysis) of the calcaneus (Figure 1(1)). In 1907, Haglund [9] described a case of heel pain during puberty associated with radiographic findings of fissuring in the apophyseal nucleus, which appeared fragmented in multiple areas and was therefore interpreted to be indicative of fractures, despite the absence of any known trauma. A few years later, in 1912, James Warren Sever first recognized that heel pain in growing children was related to repetitive microtrauma to the calcaneal apophysis—the site of insertion of the Achilles tendon—and related to a disorder of the process of endochondral ossification [10]. Later, many authors described calcaneal osteochondrosis, contributing to the body of the literature on the subject, such as Berry in 1916, Kurtz in 1917, Vulliet and Scarlini in 1920, Zaaijer in 1921, and Heine and Blenke in 1923 [7,11,12]. Due to the large number of authors who have studied the pathology, calcaneal osteochondrosis can be referred to by numerous other synonyms and historical eponyms, including calcaneal apophysitis, Wiltzer’s calcaneal osteochondritis, Lannelongue’s apophyseal osteitis, Holst and Chandrikoff’s juvenile necrotizing osteopathy, Zaaijer’s paraosteogenetic juvenilis osteopathy of the calcaneus, Müller’s calcaneal malacia, Vulliet’s disease, or, more comprehensively in terms of authorship, Haglund–Sever–Blenke disease [7].

#### 2.1.2. Epidemiology

Sever’s disease predominantly occurs in children and adolescents between 8 and 15 years of age, with sex-related differences: males have a frequency of occurrence 2 to 3 times higher than females. The typical age of onset is approximately 12 years in males and 11 years in females [13]. It accounts for 2% to 16% of musculoskeletal disorders in children and is one of the leading causes of heel pain (talalgia) in adolescents. Pain may present unilaterally or, in up to 60% of cases, bilaterally [13]. Meyerding and Stucke reported an incidence concordance in twins [14].

#### 2.1.3. Pathophysiology

Calcaneal osteochondrosis has traditionally been described as resulting from repetitive tensile stress exerted by the Achilles tendon on the calcaneal apophysis [13]. For this reason, it can be considered a traction enthesopathy that primarily involves the growth cartilage, distinct from other epiphyseal osteochondroses, such as those affecting the navicular or metatarsals, in terms of its pathogenesis. Unlike these conditions, which typically result from disruptions in the blood supply to the epiphysis, this condition is thought to stem from mechanical stress and traction forces exerted on the growth plate, leading to localized inflammation and degeneration of the cartilage.

More recently, however, magnetic resonance imaging studies have proposed a compressive rather than traction-based etiology, as metaphyseal microfractures, hemorrhage, and bone marrow edema are frequently observed. These findings suggest that compressive forces may weaken the metaphysis trabecular bone [13].

Contributing factors include Achilles tendon stiffness, the use of inadequate footwear, and equinus contracture, all of which increase mechanical stress on the posterior heel [15].

#### 2.1.4. Clinical Features

Patients typically report posterior heel pain that worsens with physical activity and improves with rest. Clinical examination often reveals Achilles tendon and/or gastrocnemius–soleus complex tightness, limited ankle dorsiflexion, and localized pain over the posterior calcaneus, sometimes accompanied by redness and swelling [16]. In some cases, medial–lateral compression of the calcaneus elicits pain, indicative of a positive calcaneal squeeze test.

#### 2.1.5. Diagnosis

**Figure 2:**
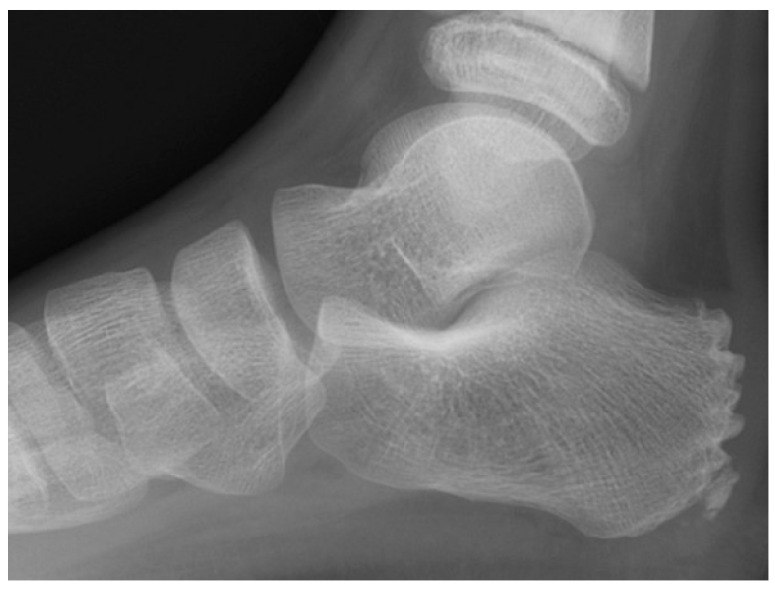
Radiograph of an 8-year-old asymptomatic male patient showing irregularity and fragmentation of the calcaneal apophysis.

**Figure 3:**
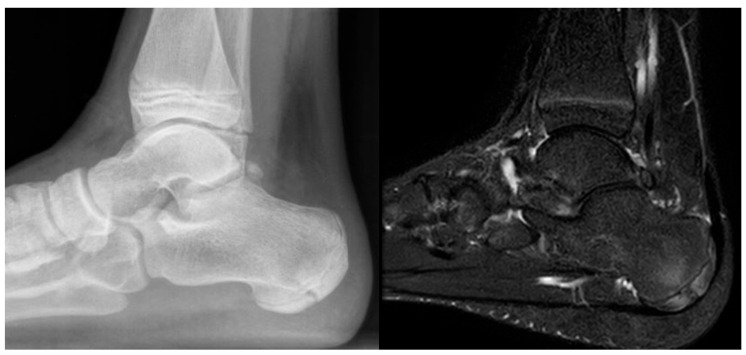
Eleven-year-old male patient. Radiograph (**left**) demonstrating typical fragmentation and bipartite appearance of the calcaneal apophysis. MRI (**right**) showing detailed fragmentation with associated bone marrow edema.

**Figure 4:**
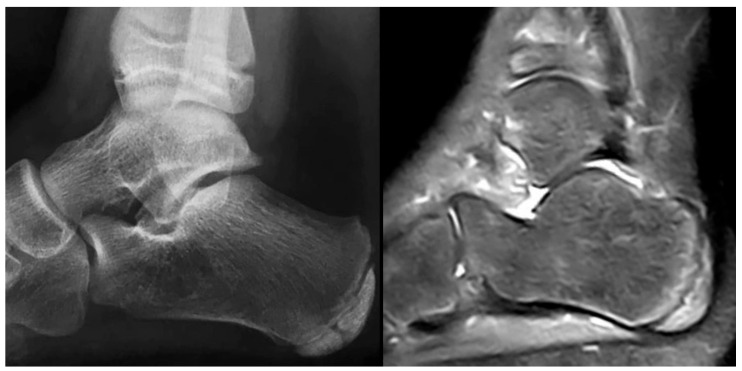
Ten-year-old male patient. Radiograph (**left**) revealing marked fragmentation, thinning, and bipartition of the calcaneal apophysis. MRI (**right**) demonstrating extensive associated bone marrow edema.

Diagnosis is based on patient history and clinical presentation. In most cases, the clinical presentation alone is adequate to establish the diagnosis, and imaging is not routinely required during the initial stages of the condition. First-line imaging studies include plain radiographs for differential diagnosis; however, they are not diagnostic, as a fragmented calcaneus can also be observed in asymptomatic children (Figure 2). The nucleus, in addition to being fragmented, may also be physiologically thickened, bipartite, smaller, or displaced upwards or downwards, without having any pathological relevance [14]. Diagnosis is further supported by magnetic resonance imaging (MRI), which can demonstrate bone marrow edema, microfractures, and stress-related bone responses [13] (Figure 3 and Figure 4).

### 2.2. Talar Osteochondrosis

#### Definition and Historical Notes

At the talus level, no pure osteochondrosis has been described, as seen in other regions, in terms of pathological anatomy and clinical and radiographic presentations (Figure 1(2)).

**Figure 5:**
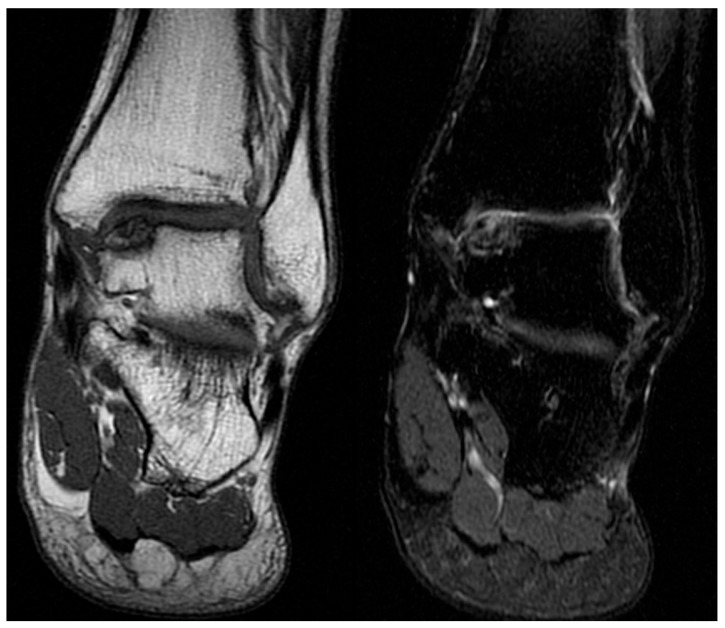
Twelve-year-old male patient presenting with pain and functional limitation in the absence of trauma. MRI reveals osteochondritis dissecans of the medial talar dome.

In rare cases, conditions of diffuse non-traumatic necrosis have been described in the developmental age [14]. Nevertheless, as the talus is a highly cartilaginous bone, with approximately 70% of its surface covered by cartilage, articulating with four bones, it is more prone to a phenomenon known as osteochondritis dissecans. This condition involves injury to the cartilage and subchondral bone (Figure 5). It was first described by Monroe in 1737, who reported the presence of a loose body in the tibiotalar joint following trauma [17]. In 1922, Kappis was the first to describe a similar lesion in the ankle joint and introduced the term “osteochondritis dissecans” to characterize cartilaginous and subchondral lesions of the tibiotalar articulation [18]. Similarly, this was reported by Diaz in 1928 [7,19].

As it does not constitute a true osteochondrosis, this condition will not be addressed in detail within this work. However, it has been included for the sake of completeness in the discussion of circulatory-related causes of foot pain, to highlight it as a potential differential diagnosis.

### 2.3. Cuboid Osteochondrosis

#### 2.3.1. Definition and Historical Notes

Osteochondrosis of the cuboid is a rare and poorly characterized condition in the literature, lacking an official eponym (Figure 1(3)). It may be confused with cuboid syndrome [20,21,22]. Historically, Silferskiöld described the first case in 1926 [23]. The first detailed study at the level of the cuboid was conducted by Koo in 1950, who reported a case in a 6-year-old Chinese boy [24].

#### 2.3.2. Epidemiology

Osteochondrosis of the cuboid is such an exceptionally rare condition that establishing a clear epidemiology is challenging, as fewer than 10 cases were described throughout the entire 20th century. Pasquali Lasagni in 1960 reviewed the literature, finding only three cases in total, attributed to Lance and Khoo [25]. Until now, no definite gender predilection has been established. The few reported cases were unilateral, but due to the lack of cases, it is not possible to make any conclusive statement on the epidemiology of the disease.

#### 2.3.3. Pathophysiology

Given the very limited number of cases described in the literature, it is difficult to establish precise etiological factors. However, trauma, infections, and developmental anomalies have been identified as contributing factors in the onset of cuboid osteochondrosis [24].

#### 2.3.4. Clinical Features

Cuboid osteochondrosis typically presents with localized pain and tenderness over the lateral midfoot, often accompanied by swelling, a limp or antalgic gait, and pain that worsens with physical activity and improves with rest [24].

#### 2.3.5. Diagnosis

In the case reported by Khoo, the patient, a 6-year-old boy, exhibited a disc-shaped flattened cuboid with moderate sclerosis and well-defined margins, consistent with osteochondrosis. Additionally, increased radiographic opacity in the tarsal region, particularly on the dorsal aspect. Follow-up radiographs taken nine months later demonstrated an increase in the size and thickness of the cuboid, although the bone remained somewhat flattened, sclerotic, and sharply demarcated [24].

### 2.4. Navicular Osteochondrosis

#### 2.4.1. Definition and Historical Notes

The osteochondrosis of the navicular bone is an avascular necrosis of the tarsal navicular, resulting from temporary interruption of its blood supply and subsequent bone necrosis and reossification (Figure 1(4)) [26]. It is also known as Köhler’s disease, as it was first described by Alban Köhler in 1908 [27]; he described the condition as “a frequent yet hitherto unrecognized disease of individual bones in children”. Later, many authors described navicular osteochondrosis, contributing to the body of the literature on the subject, such as Doviesch in the same year, Behn and Krause in 1909, Schaeffer in 1910, and Bertolotti in 1915 [7,28,29].

#### 2.4.2. Epidemiology

Navicular osteochondrosis generally affects patients of preschool age, typically between 4 and 7 years [30]. Older patients can also be affected, but involvement is rare [31]. Males are more commonly affected than females, with a male-to-female ratio ranging from 4:1 to 5:1 [30]. In most cases, the condition is unilateral; however, it may present bilaterally in approximately 20–25% of cases [32]. When bilateral, it may present asymmetrically and be associated with other forms of osteochondrosis.

#### 2.4.3. Pathophysiology

The disease is caused by a temporary avascular necrosis of the navicular bone. This condition results from a transient disruption of the blood supply, leading to ischemia and subsequent necrosis of the bone tissue. Following the necrotic phase, reparative processes ensue, ultimately restoring bone integrity. One identified risk factor is delayed ossification of the navicular (occurring at 18–24 months in females and 30–36 months in males), which renders the bone more susceptible to repetitive microtrauma and vascular compression [30]. Another possible cause may lie in anatomical variations, particularly in conditions that lead to a reduction in the space between the first cuneiform and the talus, typically caused by an increased talar prominence, which in turn increases the stresses and tensions at the level of the navicular.

#### 2.4.4. Clinical Features

The hallmark symptom of navicular osteochondrosis is localized pain, often in the midfoot, which tends to worsen with weight-bearing activities. The pain may diminish with rest but can become quite pronounced during periods of physical exertion [30].

Alongside pain, mild swelling may be observed in the affected area. The swelling is generally not severe but can be persistent. In many cases, children will exhibit signs of discomfort while walking, leading to a characteristic limp [30]. Tenderness over the navicular bone is also a common feature, and when pressure is applied to this area, the child may express sensitivity. The foot’s range of motion is often limited, particularly in the midfoot region [30]. In more advanced cases, the disease may lead to structural changes in the foot. As the navicular bone undergoes a process of degeneration, there is a possibility of bone collapse or flattening, which could result in a visible deformity of the foot. This, however, is not commonly seen and typically occurs in more severe cases [30].

#### 2.4.5. Diagnosis

**Figure 6:**
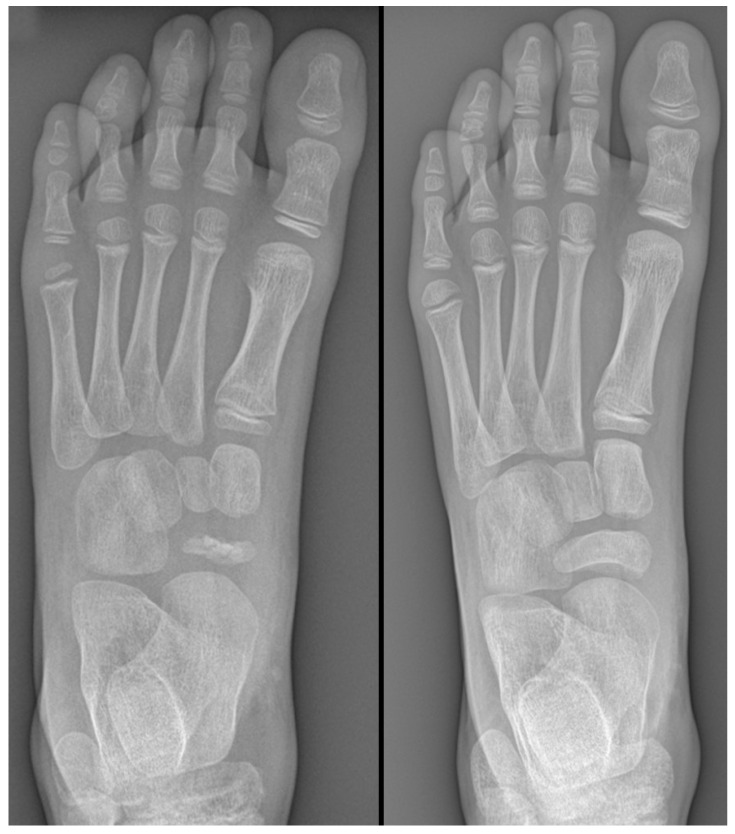
(**Left**): Radiograph of a 6-year-old male patient showing characteristic collapse and flattening of the navicular bone. (**Right**): Radiograph at 2-year follow-up after conservative treatment with non-weight-bearing casting, demonstrating good restoration of the navicular bone’s anatomical profile.

Sometimes, navicular osteochondrosis can be an incidental finding on radiographs with no focal tenderness or pain. When pain is reported, the primary diagnostic tool is radiographic imaging. Regarding the role of radiographs in the study of navicular osteochondrosis, Mouchet and Roederer wrote, “The clinical presentation is almost negligible, radiography is everything.” [33]. Initial radiographs may show signs of bone sclerosis, which is indicative of altered bone metabolism and insufficient blood supply. The radiographic findings can range from subtle changes to more evident deformities, depending on the stage of the disease. In doubtful cases, it is helpful to perform a comparative radiograph of the contralateral healthy foot. Over time, radiographs may reveal a characteristic collapse and flattening of the navicular bone. In advanced forms, the navicular becomes very thin, exhibiting a biconcave or knife-blade appearance. The radiographic appearance of the navicular takes a long time to return to normal after healing, often requiring 1 to 2 years [14] (Figure 6).

Advanced imaging techniques such as MRI may occasionally be used to further assess the extent of bone involvement or to rule out other conditions, such as infections or neoplastic processes. MRI, in particular, can provide detailed images of both bone and soft tissue and may be useful in detecting early bone marrow edema, a feature that may not be visible on plain radiographs [34].

### 2.5. Cuneiform Bones Osteochondrosis

#### 2.5.1. Definition and Historical Notes

The osteochondrosis of the cuneiform bones refers to a pathological condition characterized by the degeneration or necrosis of the subchondral bone and cartilage (Figure 1(5)) [35]. It does not have a universally established eponym; however, it is sometimes referred to as Wegner’s osteochondrosis, as Wegner was the first to describe an adult idiopathic necrosis of the medial and lateral cuneiform bones in 1928 [36]. Osteochondrosis of the intermediate cuneiform was described by Lewin in 1928 and Kuntscher in 1939 [37]. Buchmann described cases of medial cuneiform involvement in 1933 [38].

#### 2.5.2. Epidemiology

Osteochondrosis of the cuneiform bones is very rare, and consequently, no definitive epidemiological data have been established in the literature. However, Atbasi et al. conducted a review of 18 published cases, reporting an average patient age of 5.0 ± 1.3 years, with a range from 2.5 to 8 years [39]. Cuneiform involvement was more frequent on the medial and intermediate bones, while the lateral is the most rare [39]. Only one case of bilateral lateral cuneiform osteochondrosis has been reported in the literature by Mubarak [40].

According to Buchmann, the association with navicular osteochondrosis is common [14,38].

#### 2.5.3. Pathophysiology

The etiology of cuneiform osteochondrosis is unknown, but it is presumed to result from secondary necrosis due to a vascular insult leading to abnormal endochondral ossification. Risk factors may include microtrauma, rapid growth, and hormonal influences [41].

#### 2.5.4. Clinical Features

Patients typically present with pain and limping. However, asymptomatic cases have also been reported in the literature. On physical examination, localized tenderness upon palpation of the affected cuneiform bones is commonly observed.

#### 2.5.5. Diagnosis

**Figure 7:**
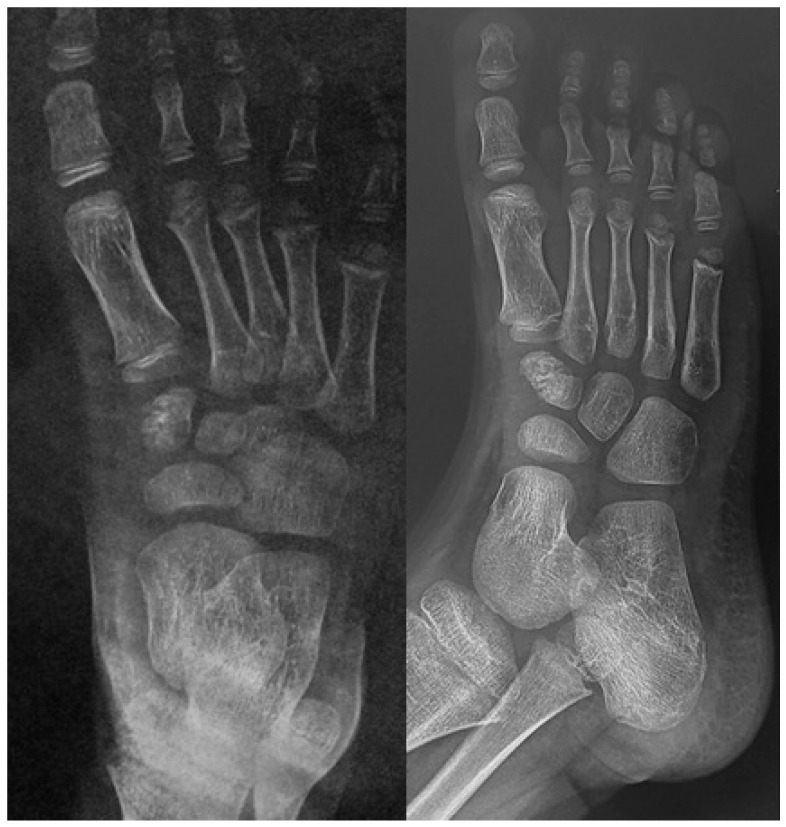
Radiograph of a 9-year-old male patient demonstrating fragmentation and irregular margins of the medial cuneiform bone.

The diagnosis is initially made based on the clinical examination. The primary diagnostic tool is radiographic imaging. Radiographic examination typically reveals a small, sclerotic cuneiform bone with irregular margins. MRI may demonstrate decreased signal intensity on T1-weighted images [41], supporting diagnosis and excluding other possible diagnoses such as tumors or osteomyelitis (Figure 7).

### 2.6. First Metatarsal Base Osteochondrosis

#### 2.6.1. Definition and Historical Notes

Osteochondrosis of the first metatarsal base refers to a pathological condition characterized by the degeneration or necrosis of the subchondral bone and cartilage (Figure 1(6)). Osteochondrosis of the first metatarsal base is also known as Grashey’s osteochondrosis, named after the one who first described the condition in 1925 and then in 1933 [42]. Subsequently, Wagner also drew attention to it in 1930 in a 6-year-old girl. Later, Breitenfelder in 1937, Karlen in 1948, and Baer in 1949 also reported on it [7].

#### 2.6.2. Epidemiology

Currently, there are very few data available in the literature regarding this condition; therefore, it is not possible to establish accurate epidemiology. However, when osteochondrosis is present, it typically affects the base rather than the head of the metatarsal, as is observed in the lesser metatarsals. This is because the distal end of the first metatarsal is not derived from the epiphysis, with the epiphysis and growth cartilage located proximally.

#### 2.6.3. Pathophysiology

The base of the first metatarsal has considerably more favorable vascular conditions compared to other sites affected by osteochondrosis. In any case, it is possible for a phenomenon to occur, for reasons that are still unknown, whereby a disruption of blood flow to the subchondral bone leads to necrosis. As the bone attempts to repair itself, it may become weakened, resulting in structural changes such as collapse or even deformity.

#### 2.6.4. Clinical Features

Osteochondrosis of the first metatarsal base often manifests with local pain, which tends to worsen during weight-bearing activities. This pain may be accompanied by swelling and tenderness around the affected joint. Patients may also experience a reduced range of motion and an altered gait. If the condition progresses, deformities or flattening of the joint may occur, further compromising foot function.

#### 2.6.5. Diagnosis

**Figure 8:**
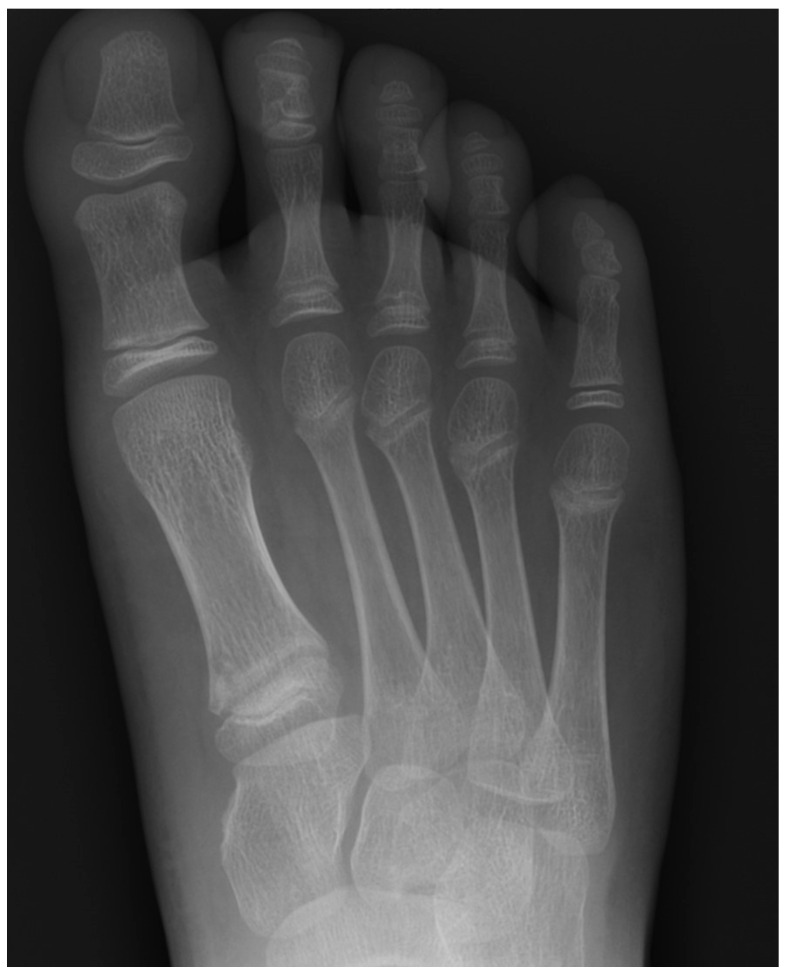
Radiograph of a 10-year-old male patient with no trauma history, showing fragmentation at the base of the first metatarsal.

Imaging studies, such as X-rays, are used to confirm the diagnosis, revealing characteristic changes such as bone sclerosis, fragmentation, or collapse (Figure 8). X-rays can also show narrowing of the joint space or other deformities. In some cases, MRI may be used to detect early bone changes or soft tissue involvement.

### 2.7. Lesser Metatarsal Head Osteochondrosis

#### 2.7.1. Definition and Historical Notes

Osteochondrosis of the lesser metatarsals is characterized by the degeneration or necrosis of the subchondral bone and cartilage at the metatarsal head level (Figure 1(7)). The first documented case of osteochondrosis affecting the lesser metatarsals was observed in the second metatarsal. Osteochondrosis of the second metatarsal head is known as Freiberg’s disease or Freiberg’s osteochondrosis, named after Alfred H. Freiberg, who first described six cases in 1914 [43,44]. In 1915, Köhler mentioned this pathological condition at the second metatarsal in his radiology textbook. Later, Köhler expanded on his studies, defining it as a disease affecting the entire joint and describing a deformity involving the base of the proximal phalanx. For this reason, osteochondrosis of the second metatarsal head is also known as Köhler II or Freiberg–Köhler disease [45]. This osteochondrosis can affect the heads of other lesser metatarsals, particularly the third, fourth, and fifth. These locations are sometimes referred to by eponyms, named after those who first described them: Panner disease, described in 1921 for the third and fourth metatarsals [46], and Mandl disease, described in 1923 for the fifth metatarsal [7].

#### 2.7.2. Epidemiology

The most affected metatarsal is the second. Osteochondrosis of the second metatarsal occurred in approximately 1 in every 2833 live births between 2000 and 2023 [47]. The peak age of onset occurs between 11 and 17 years, with cases reported into adulthood. The prevalence is significantly higher in females, accounting for an estimated 80% of cases in girls, with a female-to-male ratio of approximately 5:1 [47]. The most affected metatarsal is the second (≈68% of cases), followed by the third in about 27%, and more rarely the fourth or fifth metatarsals. The condition is typically unilateral, although bilateral involvement is observed in approximately 10% of patients [44]

#### 2.7.3. Pathophysiology

The etiology of lesser metatarsal osteochondrosis is multifactorial, characterized by repeated stress on the metatarsal head [48] leading to avascular necrosis and collapse of the subchondral bone. Contributing factors include repetitive microtrauma, vascular insufficiency due to anatomical predisposition or vascular spasm, and predisposing conditions such as a relatively long metatarsal, high levels of athletic activity, genetic factors, or systemic disorders [49,50]. Possible etiologies also include avascular necrosis following acute trauma and even tuberculosis. According to some authors, the determining factor for the onset of the disease is the changes in vascularization that occur during the peripubertal period. Between the ages of 11 and 17, the local circulation exhibits a characteristic vulnerability, as this period marks the transition in the metatarsal epiphysis from its own vascular supply to that of the diaphysis. With the disappearance of the epiphyseal cartilage, the epiphyseal and diaphyseal vessels anastomose. Simultaneously, there is a gradual reduction in the caliber of the epiphyseal vessels, with a corresponding increase in blood supply from the nutritive artery of the diaphysis. By the time adulthood is reached, blood circulation is maintained through well-established anastomoses. However, in the developmental years, this transition from one circulatory state to another has not yet been completed, and any pathogenic factor intervening during this time can disrupt local trophism. This may explain the disease’s higher prevalence in females, not so much because during this period of life, the foot is subjected to high-heeled shoes, but because in females, the fusion between the epiphysis and diaphysis occurs more rapidly than in males. Consequently, the rapid cessation of activity in the epiphyseal arteries can lead to inadequate blood supply due to insufficient anastomoses forming between these arteries and the nutritive artery of the diaphysis [14].

#### 2.7.4. Clinical Features

Lesser metatarsal osteochondrosis typically presents with metatarsalgia or forefoot pain, especially during weight-bearing activities, which improves with rest. Clinical examination often reveals localized swelling, restricted range of motion at the metatarsophalangeal joint, and the development of plantar callosities [44].

#### 2.7.5. Diagnosis

While the clinical findings can strongly suggest the presence of osteochondrosis, imaging is crucial for confirming the diagnosis and determining the extent of bone involvement.

**Figure 9:**
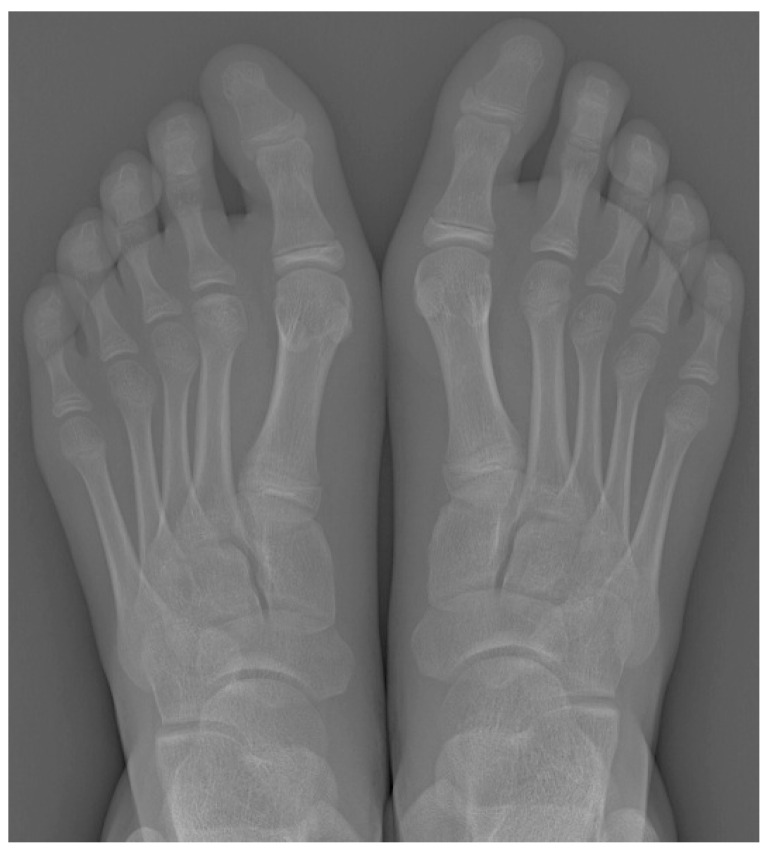
Radiograph of a symptomatic 10-year-old female patient showing early sclerosis and flattening of the second metatarsal head on the left foot, compared to the contralateral side.

Plain radiographs are the cornerstone of imaging in the diagnosis of lesser metatarsal osteochondrosis. In the early stages of the disease, when supported by a suggestive clinical presentation, obtaining a comparative radiograph of the contralateral foot can be a useful diagnostic tool (Figure 9).

**Figure 10:**
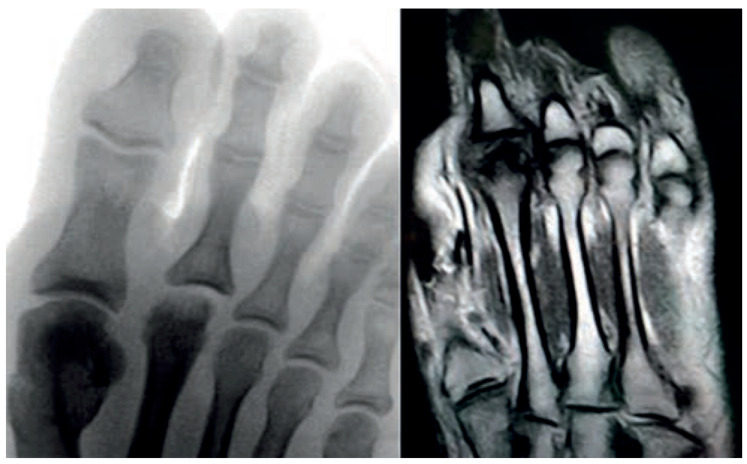
Radiograph (**left**) and MRI (**right**) of a 17-year-old female patient showing necrotic changes and initial flattening of the second metatarsal head.

**Figure 11:**
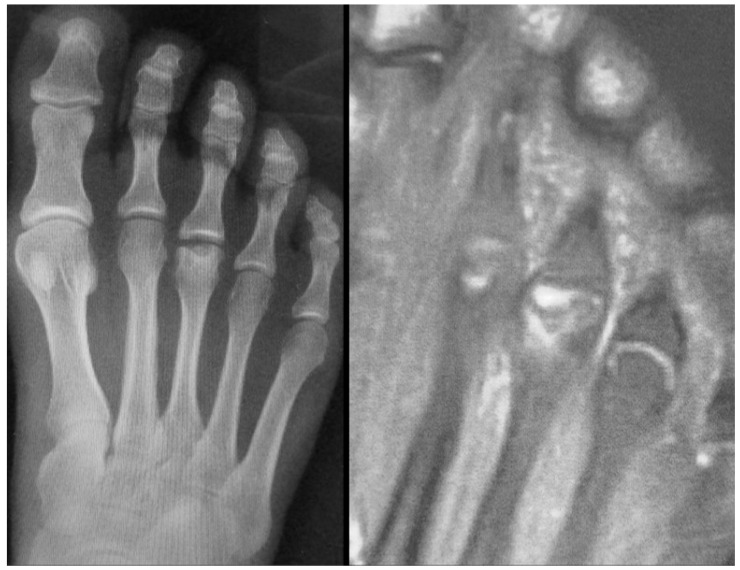
Radiograph (**left**) and MRI (**right**) of a 15-year-old male patient with osteochondrosis of the third metatarsal head and early signs of deformation.

**Figure 12:**
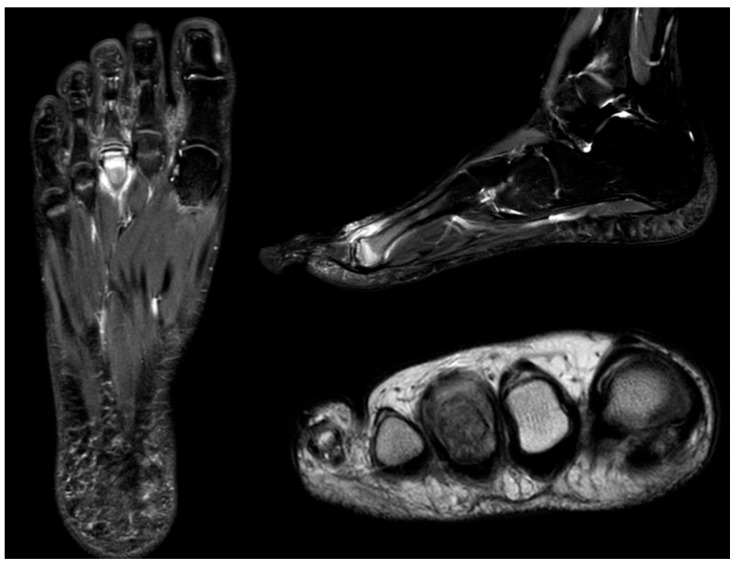
MRI of a 12-year-old female patient showing extensive bone marrow edema of the third metatarsal head, consistent with active inflammation and necrosis.

In the later stages, radiographs may reveal characteristic changes including subchondral sclerosis, fragmentation, and flattening of the affected metatarsal head (Figure 10 and Figure 11). In some cases, the presence of joint space narrowing or bony irregularities may also be observed. However, early changes, such as bone marrow edema or subtle cartilage damage, may not be visible on standard radiographs. MRI is particularly useful in the early stages of the disease, as it can detect bone marrow edema, which is a sign of active bone inflammation and necrosis, even before structural changes become apparent on radiographs (Figure 10, Figure 11 and Figure 12).

**Figure 13:**
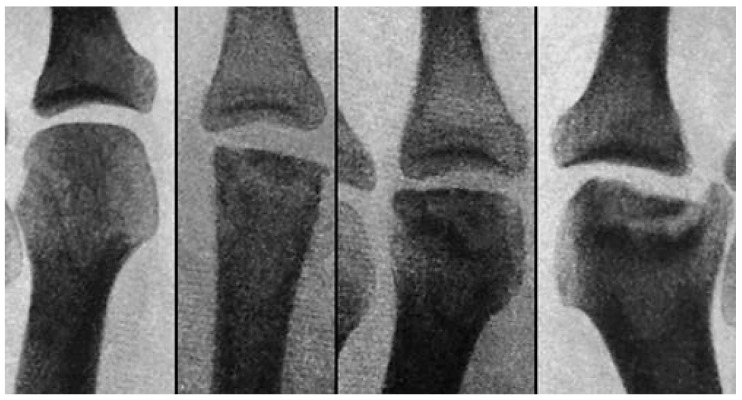
The radiographic progression of the second metatarsal head osteochondrosis in a 22-year-old female patient who had not received any treatment.

If not promptly identified and adequately treated, the condition may progress to severe deformities in adulthood (Figure 13).

### 2.8. Fifth Metatarsal Base Osteochondrosis

#### 2.8.1. Definition and Historical Notes

Fifth metatarsal base osteochondrosis is characterized by the degeneration of the subchondral bone and cartilage due to inflammation and irritation of the growth apophysis (Figure 1(8)). There is no universally accepted specific eponym, although it is often referred to as fifth metatarsal base apophysitis or as Iselin’s disease, after Iselin, who first described it in 1912 [51].

#### 2.8.2. Epidemiology

Fifth metatarsal base osteochondrosis primarily manifests in children aged 8 to 13 years. The condition is more prevalent in males. Risk factors include participation in sports such as soccer, dance, basketball, and running [52].

#### 2.8.3. Pathophysiology

Fifth metatarsal base osteochondrosis is not a true epiphyseal osteochondrosis (such as navicular or lesser metatarsals osteochondroses) but rather a traction enthesopathy involving the growth cartilage, similar to conditions like calcaneal osteochondrosis or Osgood–Schlatter disease. Fifth metatarsal base osteochondrosis is likely caused by repetitive stress exerted by the peroneus brevis tendon on the cartilaginous apophysis (which has not yet fused with the bone). This initially leads to irritation, followed by inflammation that results in pain. Symptoms typically resolve with closure of the growth center, which usually occurs by 14–15 years of age [52].

#### 2.8.4. Clinical Features

Fifth metatarsal base osteochondrosis presents with localized pain at the base of the fifth metatarsal. The pain is exacerbated by sports activities and is not associated with any specific trauma. On physical examination, palpation over the area reproduces and aggravates the pain [52].

#### 2.8.5. Diagnosis

**Figure 14:**
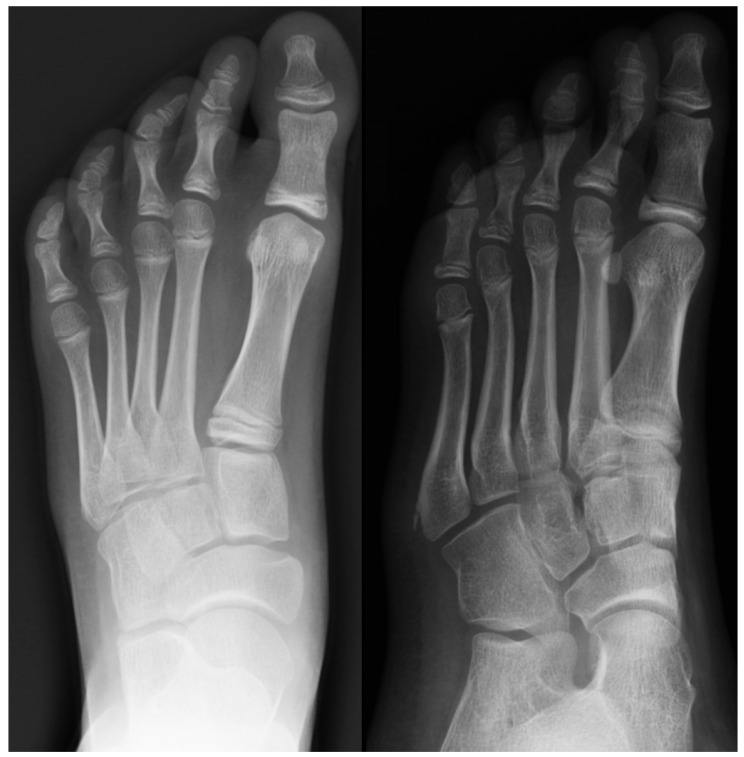
Radiograph (especially on oblique view) of an 8-year-old male patient showing fragmentation of the lateral apophysis of the fifth metatarsal base, in the absence of trauma.

The clinical findings can strongly suggest the presence of osteochondrosis. Imaging may help confirm the diagnosis. Radiographically, the condition is characterized by an elongated, irregular, or fragmented lateral apophysis. This condition is often more clearly visualized on oblique radiographic projections (Figure 14). It is often mistaken for an avulsion fracture, although in the latter, there is typically a clear history of acute trauma [52,53].

### 2.9. Sesamoid Osteochondrosis

#### 2.9.1. Definition and Historical Notes

Osteochondrosis of the sesamoid bones is characterized by the degeneration or necrosis of the subchondral bone and cartilage at the sesamoid level (Figure 1(9)) [54]. In the literature, the condition is often referred to as Renander’s disease, as the condition was first described by Axel Renander in 1924 [55]. In 1972, Ilfield and Rosen [54] described three other cases, and in 1983, Kilman et al. described six more cases, four of which were treated surgically [56].

#### 2.9.2. Epidemiology

Sesamoid osteochondrosis is such a rare condition that no epidemiological data are available in the literature.

#### 2.9.3. Pathophysiology

The pathogenesis is likely related to hypoperfusion of the sesamoid bones due to microvascular occlusion or compromised blood flow, resulting in subchondral necrosis. Chronic mechanical stress and anatomical variations in the shape and size of the sesamoids also contribute, along with rare factors such as vasculitis and coagulation disorders [57].

#### 2.9.4. Clinical Features

The most notable symptom is localized pain, which tends to be exacerbated by weight-bearing activities, or standing for prolonged periods. This pain is often sharp or aching and may worsen with specific movements, particularly those involving dorsiflexion of the big toe. Swelling and tenderness around the affected sesamoid bones are also commonly observed, particularly when pressure is applied to the area. In some cases, the affected individual may experience a feeling of instability or difficulty in performing certain foot movements due to pain and discomfort. Over time, as the condition progresses, there may be visible signs of joint stiffness and a reduced range of motion in the affected toe, making it more difficult to perform actions like pushing off during walking or running. Additionally, individuals may alter their gait to minimize discomfort, forcing the patient to walk with the foot in a supinated position to avoid loading the first metatarsal.

#### 2.9.5. Diagnosis

Radiographically, the affected sesamoid bone may appear fragmented. Native radiological changes usually become recognizable only 6 to 12 months after the onset of symptoms [54]. Diagnosis can be confirmed with MRI. Cases may be asymptomatic despite visible radiographic changes, indicating that symptoms are not always manifest. Differential diagnoses include fractures, arthritic changes, infectious sesamoiditis, and bipartite or tripartite sesamoiditis.

### 2.10. Phalangeal Osteochondrosis

#### 2.10.1. Definition and Historical Notes

The osteochondrosis of foot phalanges is characterized by the degeneration or necrosis of the subchondral bone and cartilage (Figure 1(10)). This condition is also referred to as familial osteoarthropathy of the fingers or Thiemann disease. The eponym derives from the first description by Thiemann in 1909 [58].

#### 2.10.2. Epidemiology

Osteochondrosis of foot phalanges most commonly affects the hand; involvement of the foot is exceedingly rare, with the first toe being the most frequently affected site in such cases and may involve both the proximal and distal phalanx [59]. When localized to the foot, it is considered an ultra-rare condition. A rare familial form has been described, typically with onset before the age of 25 and a benign clinical course [60]. The estimated prevalence is less than 1 per 1,000,000, classifying it as an ultra-rare disease. Sporadic cases appear to be approximately three times more common in males [61]. Osteochondrosis of foot phalanges typically manifests during adolescence and puberty, although cases have been reported in the literature ranging from as early as 4 years of age through adulthood [61].

#### 2.10.3. Pathophysiology

The pathophysiology is not fully understood, as it represents a rare condition. Histologically, it has been classified as a form of idiopathic avascular necrosis of the proximal interphalangeal joints of the hands and toes, since examinations revealed necrosis without inflammation within the affected epiphyses, often in the presence of normal vessels. It was thought to result from a transient alteration in the epiphyseal blood supply, similar to other osteochondroses. Ischemia leads to subchondral bone necrosis and fragmentation, followed by attempts at remodeling and repair [61]. A rare familial form is described with onset before 25 years of age and a benign course, and possibly some genetic risk factors could be inherited as an autosomal dominant trait [62].

#### 2.10.4. Clinical Features

On physical examination, patients present with pain and swelling of the proximal interphalangeal joints [61].

#### 2.10.5. Diagnosis

A marked discrepancy is often observed between the clinical presentation and the radiological findings. Radiographs demonstrate sclerosis, flattening, cup-shaped widening, and, in some instances, fragmentation or irregular trabecular opacity of the epiphyses. The metaphysis may appear broadened, and physeal growth can be delayed. Joint spaces usually remain preserved unless secondary arthrosis develops [59]. This condition can be misdiagnosed as juvenile rheumatoid arthritis due to overlapping clinical features. Other entities to be considered in the differential diagnosis include infection and trauma [61].

### 2.11. Osteochondrosis of Accessory Bones of the Foot

**Figure 15:**
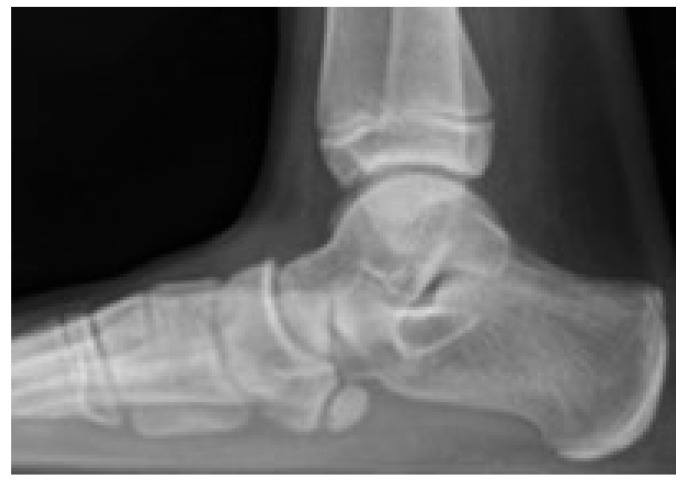
Radiograph (lateral view) of an 11-year-old female patient showing a flatfoot with a large accessory navicular bone.

In some rare cases, osteochondrosis can occur in the accessory bones of the foot, particularly those located in exposed or prominent areas. The clinical presentation and diagnosis follow the same pattern as described for the previously mentioned osteochondroses. However, the possibility of osteochondrosis in an accessory bone should always be considered in cases of unexplained localized pain. Among these are the supra-navicular bone, infra-navicular bone, second dorsal cuneo-metatarsal bone, and especially all forms of accessory navicular (Figure 1(12)). The accessory navicular generally does not ossify until 9 years of age (Figure 15). Its overall prevalence is approximately 2% to 12%. In about half of the cases, the accessory bone will go on to fuse to the navicular [63].

**Figure 16:**
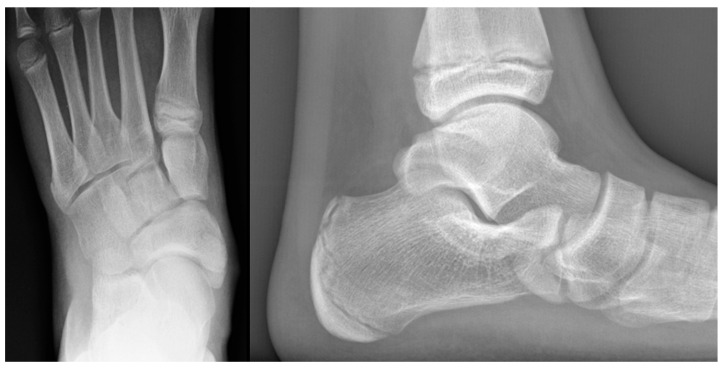
Radiograph of a 10-year-old male patient with flatfoot and a prominent accessory navicular bone.

In the case of the accessory navicular, the incidence of osteochondrosis is more frequent in females, with the most affected age group being between 12 and 15 years, especially following minor and major trauma [14]. The presence of an accessory navicular is commonly associated with flatfoot deformity (Figure 16). In many cases, a significant portion of the tibialis posterior tendon inserts onto the accessory ossicle rather than the main body of the navicular bone. This abnormal insertion may displace the tendon medially, contributing to hindfoot valgus alignment. Patients typically present with pain localized along the medial aspect of the foot. Prominence of the accessory navicular may lead to difficulties with footwear and the development of callosities. The clinical presentation may be insidious in onset or exacerbated by acute trauma to the foot or ankle. The mechanism of injury often mimics that of a medial ankle sprain, with abnormal tensile stress applied to the posterior tibialis tendon. A valgus stress event may result in fracture or separation of the ossicle from the navicular, causing instability and further contributing to the flatfoot deformity and prominence of the navicular complex. On physical examination, there may be palpable mobility of the accessory bone and marked tenderness over the medial border of the navicular. Symptoms are typically reproduced with passive forced eversion and resisted inversion of the foot.

**Figure 17:**
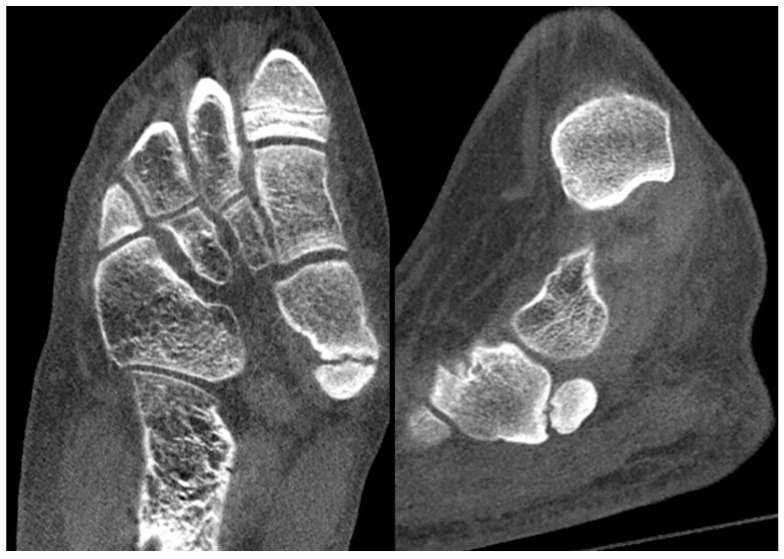
CT scan of a 12-year-old male patient showing a well-ossified accessory bone adjacent to the proximal medial margin of the navicular.

**Figure 18:**
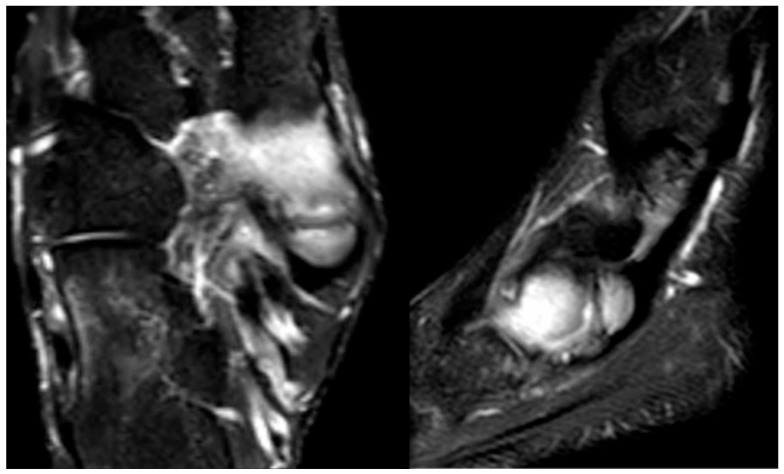
MRI of an 11-year-old symptomatic female patient showing bone marrow edema within the accessory navicular.

Standard radiographs and Computer Tomography (CT) scans commonly demonstrate a well-ossified accessory bone adjacent to the proximal medial margin of the navicular (Figure 17). In some cases, the accessory navicular is not only a source of localized symptoms but may also predispose the tibialis posterior tendon to chronic or acute injury. Tendinous fibers usually insert onto both the navicular and the accessory ossicle, and this atypical anatomy may increase the risk of tendon dysfunction, particularly in athletes. MRI in such individuals may reveal edema within the accessory navicular, indicative of chronic mechanical stress and overuse [63] (Figure 18).

## 3. Treatment

Osteochondroses are generally a self-limiting condition in all the locations investigated in the current paper. Moreover, since the various forms share the underlying etiology—a deficit in vascular supply leading to transient bone necrosis—treatment modalities are also shared. The aim of treatment is to reduce mechanical overload and stresses during the acute phase and to preserve joint congruity in order to allow for *restitutio ad integrum*. These measures are intended to prevent deformities and avoid secondary osteoarthritis.

In general, conservative treatment primarily consists of rest and especially avoidance of high-risk activities, application of ice, and the use of non-steroidal anti-inflammatory drugs for pain relief [64]. Surgery remains uncommon, and most patients can be managed with rest, offloading, and sometimes immobilization in a short leg plaster cast. Rest and immobilization times vary depending on the symptoms and location of osteochondrosis. In the osteochondrosis of the base of the fifth metatarsal, the period of immobilization can range from 2 to 4 weeks [65]. In the osteochondrosis of the navicular bone, some authors recommend immobilization for 3 to 6 weeks [63]. Similarly, in Freiberg’s disease, it is recommended to keep the cast on for 6 weeks [66]. Finally, osteochondrosis of the calcaneus is the one that requires the longest time, as some authors recommend from 6 to 12 weeks [67].

In Freiberg’s disease, after an appropriate period of unloading (3–6 weeks), weight bearing can be resumed using dedicated shoes (with a special heel that prevents weight-bearing on the forefoot), or a stiff-soled shoe with a rocker bottom can be helpful to offload the forefoot during toe-off. Alternatively, orthotics with a metatarsal pad or bar can be used to relieve pressure under the metatarsal heads [66].

As previously discussed, traction apophysitis—such as calcaneal and fifth metatarsal base osteochondroses—is characterized by a different etiology. In these cases, reducing the strain on the enthesis represents an effective treatment. To achieve this goal, several treatment modalities are available, firstly, rest—as with other forms of osteochondrosis—followed by physical therapy and, when needed, orthoses or immobilization in a cast.

In the context of calcaneal osteochondrosis, physical therapy mainly consists of eccentric calf strengthening and reinforcement exercises, while heel raises or heel cups are commonly recommended to reduce tension on the Achilles tendon [68]. Wiegerinck et al. conducted a study comparing activity cessation, heel raise inlay, and physical therapy, which all results in a clinically relevant and statistically significant pain reduction [67].

Regarding osteochondrosis of the base of the fifth metatarsal, physical therapy mainly consists of an exercise program with emphasis on strengthening, stretching, and proprioception (stabilization and coordination skills) of the foot and ankle [63]. Foot orthosis with a small lateral elevation or a lateral wedge ensures reduced stress on the peroneal muscles during daily activities, such as walking and running, and has been proven to be beneficial [69].

Acute surgical intervention is no longer considered. In the past, percutaneous perforations were performed at the level of the growth plates, but this technique has been abandoned over the years [14,33]. The only indication for acute treatment may be the removal of a painful accessory navicular, which, in children, is frequently associated with flatfoot, which may require concurrent treatment. Surgical intervention for osteochondroses is primarily indicated in adult patients who present with disabling sequelae resulting from residual deformity or secondary osteoarthritic changes. In such cases, the choice of surgical treatment is guided by established algorithms and is tailored to the specific pathological anatomy and functional impairment observed.

## 4. Conclusions

Foot osteochondroses represent a spectrum of self-limiting developmental conditions affecting the pediatric population. Conservative treatment remains the cornerstone of management, with long-term outcomes typically favorable. However, early diagnosis and appropriate intervention are critical to achieving optimal results. It is the authors’ hope that the overview provided in this paper will enhance clinical awareness, support timely and accurate diagnosis, and ultimately help prevent misdiagnosis, secondary deformities, and the progression to early osteoarthritis.

## Data Availability

The data presented in this study are available upon request from the corresponding author; however, restrictions apply to the availability of these data, which were used under license for this study, and they are thus not publicly available.
